# The Era of Immunotherapy in Small-Cell Lung Cancer: More Shadows Than Light?

**DOI:** 10.3390/cancers15245761

**Published:** 2023-12-08

**Authors:** Sabrina Rossi, Arianna Pagliaro, Angelica Michelini, Pierina Navarria, Elena Clerici, Davide Franceschini, Luca Toschi, Giovanna Finocchiaro, Marta Scorsetti, Armando Santoro

**Affiliations:** 1 Medical Oncology and Hematology Unit, IRCCS Humanitas Research Hospital, Via Manzoni 56, Rozzano, 20089 Milan, Italy; arianna.pagliaro@humanitas.it (A.P.); angelica.michelini@humanitas.it (A.M.); luca.toschi@cancercenter.humanitas.it (L.T.); giovanna.finocchiaro@humanitas.it (G.F.); armando.santoro@humanitas.it (A.S.); 2 Department of Biomedical Sciences, Humanitas University, Via Rita Levi Montalcini 4, Pieve Emanuele, 20072 Milan, Italy; marta.scorsetti@hunimed.eu; 3 Department of Radiotherapy and Radiosurgery, IRCCS Humanitas Research Hospital, Via Manzoni 56, Rozzano, 20089 Milan, Italy; pierina.navarria@humanitas.it (P.N.); elena.clerici@humanitas.it (E.C.); davide.franceschini@humanitas.it (D.F.)

**Keywords:** small-cell lung cancer, immunotherapy, radiotherapy

## Abstract

**Simple Summary:**

Small-cell lung cancer is the most aggressive form of lung neoplasia, treated in recent decades with chemotherapy alone. In the last few years, the advent of immunotherapy has changed the landscape in the treatment of non-small-cell lung cancer, and in small-cell lung cancer as well. However, the effectiveness of immunotherapy and the potential predictors of the response are still not completely established. This review aims to investigate the current knowledge in this field.

**Abstract:**

Small-cell lung cancer is an extremely chemo-sensitive disease; the addition of immunotherapy to chemotherapy has demonstrated a slight clinical benefit in pivotal trials, even with a statistically significant difference in terms of survival outcomes when compared to chemotherapy alone. In this scenario, the role of radiotherapy as a consolidation treatment in thoracic disease or as a prophylactic therapy in the brain should be clarified. In addition, due to the frailty and the poor prognostic characteristics of these patients, the need for predictive biomarkers that could support the use of immunotherapy is crucial. PD-L1 and TMB are not actually considered definitive biomarkers due to the heterogeneity of results in the literature. A new molecular classification of small-cell lung cancer based on the expression of key transcription factors seems to clarify the disease behavior, but the knowledge of this molecular subtype is still insufficient and the application in clinical practice far from reality; this classification could lead to a better understanding of SCLC disease and could provide the right direction for more personalized treatment. The aim of this review is to investigate the current knowledge in this field, evaluating whether there are predictive biomarkers and clinical patient characteristics that could help us to identify those patients who are more likely to respond to immunotherapy.

## 1. Introduction

Small-cell lung cancer (SCLC) is the most aggressive form of lung neoplasia, strongly associated with cigarette smoking [1]. The vast majority of patients present metastatic spread since the time of diagnosis, and the median overall survival (mOS) without treatment is dramatically poor, ranging between 2 and 4 months [1].

According to the Veterans Administration Lung Study Group (VALSG), limited-stage small-cell lung cancer (LS-SCLC) is defined as a disease confined to one hemithorax that can be encompassed within a reasonable radiation field, while extensive-stage small-cell lung cancer (ES-SCLC) is described as a widespread disease that is greater than a radiation field [2].

SCLC is an extremely chemo-sensitive disease, and, nowadays, the standard treatment for ES-SCLC is platinum-based chemotherapy, in association with etoposide, which has demonstrated rapid responses but poor long-term outcomes, with only 7% of patients alive at 2 years [3]. Nevertheless, the advent of immunotherapy has represented a breakthrough in the field of thoracic oncology, and the approval of both atezolizumab and durvalumab in association with chemotherapy as a first-line therapy in ES-SCLC has increased the available treatment armamentarium [4]. Atezolizumab in combination with platinum-based chemotherapy as a first-line treatment in ES-SCLC has been approved by the FDA and EMA based on the results of IMpower133, a phase III trial that randomized patients to receive either carboplatin, etoposide and atezolizumab or carboplatin, etoposide and a placebo [5,6,7]. Conversely, durvalumab has been endorsed in light of the phase III CASPIAN trial, a three-arm study where patients were randomized to receive either platinum plus etoposide, and durvalumab with or without tremelimumab, or platinum plus etoposide alone [8]. Both trials yielded a statistically significant improvement in overall survival (OS) and showed a good safety profile, establishing a new standard of care [7,8].

Similarly, in the phase III Checkmate 451 trial, the efficacy of nivolumab with or without ipilimumab versus a placebo in ES-SCLC patients with an ongoing response after four cycles of platinum-based chemotherapy was explored. However, the primary endpoint of OS was not met, and nivolumab monotherapy did not demonstrate an ability to prolong OS compared with the placebo [9].

Firstly, the role of immunotherapy was investigated in previously treated ES-SCLC patients. The efficacy of nivolumab alone and in combination with ipilimumab was explored in the phase 1/2 Checkmate 032 trial: in the randomized cohort of the study, nivolumab monotherapy demonstrated a 24-month OS rate of 17.9% and a longer duration of response [10]. Based on these results, the FDA approved nivolumab as a third-line treatment in 2018 [11]. Similarly, pembrolizumab received FDA approval in the same setting in 2019 [12], after exhibiting a 2-year OS rate of 20.7% and an objective response rate (ORR) of 19.3% in the phase Ib KEYNOTE 028 and the phase II KEYNOTE 158 trials [13]. Notwithstanding this, both pembrolizumab and nivolumab failed to show an OS improvement in the corresponding phase III trials and, in 2020/2021, their marketing authorization was withdrawn [14,15]. Despite the era of precision medicine, the treatment of ES–SCLC remains a challenge for oncologists who only have few options of treatment. Hitherto, in this subgroup of patients, no prospective trial comparing the efficacy of the available immune checkpoint inhibitors (ICIs) is conducted. The aim of this review is to better investigate the efficacy of immunotherapy in ES-SCLC, identifying biomarkers that may be able to predict the treatment response.

## 2. Immunotherapy for First-Line Treatment of ES-SCLC

After decades of minimal progress in treating ES-SCLC, the use of ICIs has provided significant improvements in OS and progression-free survival (PFS). The first ICI to demonstrate a survival benefit in addition to etoposide–platinum (EP) in treatment-naïve ES-SCLC was atezolizumab in the IMpower133 phase III trial [7]. Notably, the addition of atezolizumab to the standard treatment showed a significant survival benefit, with a median OS of 12.3 months for the atezolizumab arm vs. 10.3 months for the placebo arm (hazard ratio (HR) for death: –0.70; 95% confidence interval (CI): 0.54–0.91; *p* = 0.007), and a median PFS of 5.2 vs. 4.3 months, respectively (HR: 0.77; 95% CI: 0.62–0.96, *p* = 0.02) [7]. With regard to the baseline patient characteristics, 8.5% (17 out of 201) and 38.3% (77 out of 201) of patients in the atezolizumab group and 8.9% (18 out of 202) and 35.6% (72 out of 202) in the placebo group had brain and liver metastases, respectively. Among the patients with brain metastases (BMs) at baseline, there was no significant difference in the median OS between the experimental and control arms (HR: 0.96, 8.5 vs. 9.7 months, respectively, 95% CI: 0.46–2.01), probably due to the small sample size of this subgroup [7,16]. On the other hand, the addition of atezolizumab in patients with liver metastases seems to confer only a modest benefit in terms of the median OS (HR: 0.75, 9.3 months vs. 7.8 months, respectively, 95% CI: 0.52–1.07), despite this subgroup representing more than one third of the patients included [16].

Durvalumab was the second ICI approved for ES-SCLC due to the results of the CASPIAN trial [8]. A survival analysis after 3-year follow-up evidenced that the addition of durvalumab to chemotherapy (both cisplatin and carboplatin) demonstrated a longer median OS (12.9 vs. 10.5 months in chemotherapy arm; HR: 0.71; 95% CI: 0.60–0.86, *p* = 0.0003). A sustained clinical benefit was also observed in PFS, with a PFS rate at 12 months of 18% in the durvalumab arm and 5% in the control arm. The addition of tremelimumab to durvalumab and chemotherapy was associated with a statistically significant improvement in OS when compared to chemotherapy alone (HR: 0.81, 95% CI: 0.67–0.97, *p* = 0.02) with 36-month OS rates of 15.3% in the durvalumab + tremelimumab + chemotherapy arm versus 5.8% with chemotherapy alone (but with a median OS of 10.4 vs. 10.5, respectively) [8]. With regard to the baseline patient characteristics, 10% (28 out of 268) and 40% (108 out of 268) of patients in the durvalumab + EP group and 10% (27 out of 269) and 39% (104 out of 269) of patients in the control arm had brain and liver metastases, respectively. Notably, in this trial, only the EP arm could receive prophylactic cranial irradiation (PCI), which was not permitted in the durvalumab + EP arm [8]. The HRs for OS consistently favored durvalumab + EP vs. EP across all subgroups, including patients with liver (HR: 0.87, 95% CI 0.66–1.16) and brain (HR: 0.76, 95% CI: 0.43–1.33) metastases, despite PCI not being allowed in the experimental arm [8,17].

In the Keynote-604 trial, the addition of pembrolizumab to EP (both cisplatin and carboplatin) showed a statistically significant improvement in median PFS compared to the placebo + EP (4.5 vs. 4.2 months, respectively, HR: 0.75; 95% CI: 0.61–0.91; *p* = 0.0023), while only a clinical prolongation in OS was highlighted (HR, 0.80; 95% CI: 0.64–0.98; *p* = 0.0164) [18]. With regard to the baseline patient characteristics, in the pembrolizumab + EP arm, 14.5% (33 out of 228) and 41.7% (95 out of 228) had brain and liver metastases, respectively; in the control arm, only 9.8% (22 out of 225) and 40.9% (92 out of 225) of patients had these metastases, respectively [18]. Notably, PCI was allowed in both arms in patients achieving a complete (CR) or partial response (PR). The HRs for OS favored pembrolizumab in patients with liver metastases (HR: 0.75, 95% CI: 0.55 to 1.02), but not patients with BMs at baseline (HR: 1.32, 95% CI 0.72 to 2.42) [18].

The results of IMpower133, CASPIAN and Keynote 604 demonstrated that the addition of immunotherapy to EP provides a significant survival benefit in patients affected by ES-SCLC. However, it is worth noting some differences in the study results and design. Firstly, the IMpower133 trial allowed only the use of carboplatin, while in both the CASPIAN and Keynote–604 trials, both cisplatin and carboplatin were permitted [7,8,18]. In patients with asymptomatic or treated BMs at baseline, the results are still controversial. In the IMpower133, CASPIAN and Keynote–604 trials, a small proportion of patients with baseline BMs were enrolled (≈10%) [7,8,18]. Nevertheless, PCI was permitted only in the control arm in the CASPIAN trial, while in IMpower133 and Keynote-604, it was permitted in both the experimental and control arms [7,8,18]. Based on the results of IMpower133 and Keynote–604, patients with baseline BMs did not benefit from the addition of atezolizumab or pembrolizumab to EP, while in the CASPIAN trial, the HR for OS favored the addition of durvalumab to EP even in this subset of patients, although the observed benefit seemed to be minimal [7,8,18]. Furthermore, considering patients with liver metastases, durvalumab, atezolizumab and pembrolizumab seemed to confer only a minimal survival benefit when compared to the EP arm [8,16,18].

Furthermore, it is worth noting that the efficacy of immunotherapy in patients with ES-SCLC has been also proven in three other phase III Asian trials. Particularly, the addition of either adebrelimab, serplulimab or tislelizumab to EP showed a benefit in OS (15.3 vs. 12.8 months, HR: 0.72, *p* = 0.0017, 95% CI: 0.58–0.90; 15.5 vs. 13.5 months, HR: 0.75, *p* = 0.0035, 95% CI: 0.61–0.92; and 15.4 vs. 10.9 months, HR: 0.63, *p* < 0.001, 95% CI: 0.49–0.82, respectively), endorsing the hypothesis that immunotherapy may play a role in patients suffering from SCLC [19,20,21]. Data from these trials are reported in Table 1.

However, despite these positive results, the addition of ICIs to platinum-based chemotherapy confers only a modest prolongation of OS, although there is a subgroup of patients that seems to be more likely to respond to maintenance ICIs, whose characteristics need to be better identified.

As a matter of fact, real-world patients have poorer prognostic characteristics, resulting in worse survival outcomes [22]. In the CANTABRICO trial, the efficacy and safety of durvalumab + EP were assessed in a real-world ES-SCLC population in Spain (101 patients from 35 sites included) [23]. More than half of patients (56%) were ≥65 yrs old, 34% of patients had liver metastases and 11% has BMs. According to preliminary data, the median PFS was 6.1 months, consistent with the results of CASPIAN, and notable differences were found in PFS according to the number of cycles of induction chemotherapy received (≤4 cycles: 5.4 m, >4 cycles: 6.9 m, *p* = 0.010) [23]. Similarly, the MAURIS trial aimed to evaluate the efficacy and safety of atezolizumab + EP in a patient population closer to clinical practice (i.e., patients with a PS ECOG 2 and/or untreated asymptomatic BMs) and—according to the interim analysis—PFS and ORR seem to be aligned to those of IMpower133 [24].

## 3. Ongoing Trial on Immunotherapy in Limited- and Extensive-Stage SCLC

The role of immunotherapy is also currently under evaluation in limited-disease small-cell lung cancer (LD-SCLC). As a matter of fact, in limited-stage non-small-cell lung cancer (LS-NSCLC), the benefit exhibited in the PACIFIC trial by consolidation durvalumab after concurrent chemo-radiotherapy (cCT-RT), with a median OS of 47.5 months and a median PFS of 16.9 months, has paved the way to the study of ICIs’ efficacy in LD-SCLC [25]. In this setting, the phase II STIMULI trial aimed to assess the activity of nivolumab plus ipilimumab vs. observation after the completion of cCT-RT and prophylactic cranial irradiation [26]. However, with only 153 patients enrolled, the study was closed prematurely due to slow accrual. At the data cut-off, there was no statistically significant improvement in either PFS with immunotherapy compared with a placebo (10.7 vs. 14.5 months, HR = 1.02; 95% CI: 0.66–1.58; stratified log-rank *p* = 0.93), or OS (HR = 0.95; 95% CI: 0.59–1.52; stratified log-rank *p* = 0.82) [26].

Another study evaluating consolidation immunotherapy in LS-SCLC is the phase III ADRIATIC trial, which randomized patients to receive durvalumab with or without tremelimumab vs. a placebo after the completion of cCT-RT +/− PCI and no evidence of disease progression (PD) [27]. The combination of durvalumab plus tremelimumab has indeed demonstrated antitumor activity and a good safety profile in pretreated ES-SCLC in a series of early-phase trials, also suggesting a possible beneficial role in a localized setting [27]. The primary endpoints are OS and PFS, and the estimated primary completion date is September 2024. Similarly, the ongoing phase II ACHILES study (NCT03540420) is investigating the benefit of atezolizumab after cCT-RT; the primary endpoint is the 2-year survival rate, and the estimated primary completion date is December 2023.

The benefit shown of consolidation radiotherapy in patients affected by ES-SCLC who have not progressed after the standard platinum-based chemotherapy, together with the recent results obtained for immunotherapy, have led researchers to also evaluate new treatment strategies in this setting. Particularly, the phase II/III RAPTOR trial is actively accruing patients to test whether the addition of consolidative radiotherapy to maintenance immunotherapy can improve survival outcomes in patients without disease progression after chemotherapy [28]. Among the new ICIs under investigation, it is worth mentioning tiragolumab, which is an anti-TIGIT (T-cell immunoglobulin and ITIM domain) drug that has demonstrated strong anti-tumor activity in a phase Ia/Ib trial, with an ORR of 50% and a disease control rate (DCR) of 79% in the NSCLC cohort [29]. These results have been confirmed in the phase II CITYSCAPE study, which randomized chemotherapy-naive stage IV NSCLC patients with a PD-L1 TPS ≥1% to receive atezolizumab plus tiragolumab or a placebo. The combination of atezolizumab and tiragolumab has shown a statistically significant improvement in the ORR (37% vs. 21%) and the PFS (5.6 vs. 3.9 months), together with an increase in survival outcomes, with an OS of 23.2 vs. 14.5 months [30]. Based on these results, tiragolumab in combination with atezolizumab was granted breakthrough therapy designation (BTD) by the FDA in 2021 as first-line treatment of metastatic PD-L1-high NSCLC patients without epidermal growth factor receptor (EGFR) or anaplastic lymphoma kinase (ALK) mutations [31]. These promising findings have also boosted research on tiragolumab in ES-SCLC with phase III SKYSCRAPER-02 (NCT04256421). In this trial, untreated ES-SCLC patients were randomly assigned to receive atezolizumab plus carboplatin and etoposide either with or without tiragolumab. In the interim analysis of 2022, no benefit was seen of the addition of tiragolumab to atezolizumab, with a median PFS of 5.4 months with tiragolumab and 5.6 months with a placebo and a median OS of 13.6 months for both cohorts [32]. This was confirmed in the subgroup analyses, which did not reveal any advantage in the different subgroups [33]. SKYSCRAPER-02 will continue with the follow-up until the planned primary OS analysis. Ongoing trials for LS-SCLC are summarized in Table 2.

Furthermore, the phase Ib trial DeLLphi-303 (NCT05361395) is investigating the first-line combination of tarlatamab with carboplatin, etoposide and anti–PD-L1 in ES-SCLC. Tarlatamab is a bispecific T-cell engager molecule (HLE BiTE), which directs T cells to cancer cells expressing delta-like ligand 3 (DLL3, expressed in 85–94% of patients with SCLC), leading to their lysis [34]. The primary objective is to evaluate the safety and tolerability, and determine the recommended phase 2 dose and/or maximum tolerated dose of tarlatamab in combination with an anti–PD-L1 with or without chemotherapy, and the estimated primary completion date is December 2025.

The phase II DeLLphi-301 trial investigated the efficacy of two different doses of tarlatamab (10 mg and 100 mg) in patients with previously treated ES-SCLC [35]. The 10 mg dose was selected for subsequent tarlatamab studies due to its more favorable benefit-to-risk profile, showing an ORR of 40% (97.5% CI: 29–52) and a mOS of 14.3 months (95% CI: 10.8—not evaluable). These findings are encouraging relative to the outcomes of current second-line treatment options, like lurbinectedin (ORR: 35%, mOS: 9.3 months) and topotecan (ORR: 17%, mOS: 7.8 months) [36,37]. Notwithstanding, a longer follow-up will give us more information about the long-term survival and the durability of response.

## 4. Future of Radiotherapy Treatment in Patients with LS-SCLC and ES-SCLC

Historically, patients affected by ES-SCLC have a significant risk of thoracic recurrence after first-line chemotherapy. It was estimated that almost 75% of patients still have residual disease in the thorax after chemotherapy, and in approximately 90% of them, disease will progress at this site [38]. Based on these assumptions, four randomized trials, summarized in Table 3, evaluated the effectiveness of thoracic radiotherapy as consolidation treatment [39,40,41,42]. Despite this scientific effort, the role of thoracic radiotherapy (RT) remained uncertain, and the results were unconvincing. When RT is performed, doses usually range between 30 and 45 Gy in 10–15 fractions, although retrospective data suggest that a dose ≥45 Gy is correlated with better survival outcomes [43,44]. All these uncertainties about the effectiveness of RT recently increased since the introduction of immunotherapy as a maintenance treatment after first-line platinum-based chemotherapy. The results of immunotherapy trials are presented above. Regarding RT, both the IMpower 133 and CASPIAN trials did not allow RT unless for palliative purposes. It is noteworthy that in both trials, the relapse rates remained quite high, with a median PFS of 5.2 and 5.1 months in the IMpower-133 and CASPIAN trials, respectively [7,8]. The patterns of recurrence were not described; however, it is reasonable to suppose that intrathoracic relapse was the most common site of recurrence. The hypothesis that consolidative RT in combination with immunotherapy could contribute to reducing the recurrence rate is actually under evaluation. The ongoing NRG LU007 (NCT04402788) will provide crucial answers in this field. Furthermore, considering the expanding and convincing role of stereotactic RT for oligometastasis ablation in most of solid tumors [45,46], another possible issue could regard the role of radiotherapy limited to residual thoracic disease, instead of the whole-body consolidation of residual disease. Although data on SCLC are extremely limited and retrospective, future studies should try to investigate this topic. A recent American consensus still considers thoracic radiation (30–54 Gy) as an appropriate treatment for patients undergoing first-line chemoimmunotherapy [47]. Notably, SCLC patients have a high risk of developing BMs, up to 80% at 2 years [48]. The role of PCI in ES-SCLC patients was recognized by the phase 3 EORTC trial, with a significant prolongation of overall survival in the PCI group (6.7 vs. 5.4 months in control arm; *p* = 0.003) [49]. Nevertheless, complete brain imaging before randomization was not mandatory and patients with asymptomatic BMs could be enrolled. In 2017, the results of a multicenter randomized phase 3 trial, comparing PCI and MRI surveillance, demonstrated a reduction in BM rate in the PCI group, but not an increase in OS [50]. To date, in the chemoimmunotherapy era, the role of PCI in ES-SCLC without progression after systemic therapy remains a matter of debate. In the IMpower 133 trial, 10.9% of patients in both arms underwent PCI, and in the CASPIAN trial, only 8.3% patients in the chemotherapy arm received PCI, while it was not allowed in the experimental arm [8]. In both trials, the time to BM appearance was longer in the chemoimmunotherapy arm, thus suggesting that immunotherapy may retard brain progression [51,52]. Nowadays, recent Canadian consensus recommendations, and the European Society for Medical Oncology (ESMO) and the American Society for Radiation Oncology (ASTRO) guidelines, strongly recommended a radiation oncologist evaluation for PCI or magnetic resonance surveillance [53,54].

Historically, in LS-SCLC disease in complete remission after first-line treatment, PCI significantly improved the OS outcome. This indication is based on a meta-analysis of seven prospective studies, published in 1999, but at that time, only a few of the patients enrolled had a baseline brain MRI [55]. Recently, a meta-analysis of 28 retrospective studies, for a total of 18,575 patients, confirmed the advantage of OS in the PCI group, as well as in patients treated with chemoradiotherapy [56]. Therefore, in LS-SCLC without progression after chemoradiotherapy, PCI is recommended. In selected cases (stage I, age >70 years or frail patients), the omission of PCI should be discussed with the patient [53,54]. The standard dose of PCI is 25 Gy in 10 daily fractions [57]. Its neurocognitive toxicity has been well documented in the past; hippocampal avoidance (HA) PCI should be a choice to reduce the risk of these deficits [58,59]. In 2021, two phase 3 randomized trials, comparing HA-PCI versus PCI, reported discordant results for the reduction in neurocognitive deficits, without any difference in OS or the BM control rate [60,61]. For this reason, the results from the NRG CC003 trial, comparing HA-PCI versus PCI with the use of memantine and the central revision of RT plans, will probably answer these unsolved questions [62].

A challenging issue is the use of stereotactic radiosurgery (SRS) in SCLC BMs. Nowadays, SRS is the standard for limited BMs in NSCLC, defined as total metastatic volume less than 15 cc [63,64,65]. The widespread use of brain MRI, the integration of immunotherapy in systemic treatments and the controversies about PCI have increased the interest in SRS for BMs in SCLC patients. The FIRE-SCLC Cohort Study analyzed 710 patients affected by ES-SCLC treated with SRS for BMs. The median OS was 11.0, 8.7 and 8.0 months for patients with one lesion, two to four lesions and five to ten lesions, respectively. A propensity score-matched analysis comparing = SRS with WBRT was performed. In the WBRT group, an improved time to central nervous system progression was demonstrated, without an OS benefit [66]. A recent review including 31 studies and a meta-analysis of 7 retrospective studies reported a longer OS in SCLC patients with BMs treated with SRS rather than WBRT [67]. Based on this background, several studies are ongoing (Table 4).

## 5. SCLC Molecular Subtypes and Response to Immunotherapy

Despite the increasingly personalized approaches for the treatment of patients with NSCLC, SCLC is still treated as a single and monolithic entity. Historically, SCLC was divided into two broad categories: a classic neuroendocrine (NE) subtype and a variant non-neuroendocrine (non-NE) subtype [68]. To improve the treatment outcomes of ES-SCLC, Rudin et al. recently defined four major subtypes of SCLC based on the expression of the following key transcription factors: achaete-scute homolog 1 (ASCL1), neurogenic differentiation factor 1 (NEUROD1), yes-associated protein 1 (YAP1) and POU class 2 homeobox 3 (POU2F3), which correspond to the SCLC-A (ASCL1-dominant), SCLC-N (NEUROD1-dominant), SCLC-P (POU2F3-dominant) and SCLC-Y (YAP1-dominant) molecular subtypes, respectively [69]. Particularly, ASCL1 and NEUROD1 are the two master transcription factors that govern NE differentiation, while POU2F3 and YAP1 govern non-NE differentiation [70]. Despite this classification, subsequent immunohistochemical (IHC) analyses have failed to confirm a unique YAP1 subtype [71]. In light of these findings, using tumor expression data and non-negative matrix factorization (NMF), Gay et al. identified four SCLC subgroups defined by the differential expression of transcription factors ASCL1, NEUROD1 and POU2F3 or the low expression of all three transcription factor signatures accompanied by an inflamed gene signature (SCLC-A, N, P and I, respectively) [72]. The clinical implications of this classification are significant, since each subtype demonstrates specific vulnerability to different therapies. SCLC-I is a novel, mesenchymal and inflamed SCLC subtype, recently proposed to replace the YAP1 subtype. It is worth noting that SCLC-I tumors are characterized by high expression of CD274, which encodes PD-L1; PDCD1, which encodes PD-1; and CTLA4, CD80 and CD86, which encode the ligands for CTLA4 [72]. On the basis of a retrospective analysis of the tumor transcriptome data from the IMpower133 trial, the HRs for OS favored atezolizumab + EP across all four SCLC subtypes, with a modest trend toward improved OS in SCLC-I [72]. Furthermore, a subtype-by-subtype analysis comparing survival benefit between atezolizumab + EP and a placebo + EP highlighted that the addition of atezolizumab resulted in survival prolongation, especially in the SCLC-I subtype. Conversely, the addition of atezolizumab to EP resulted in only a modest gain in median OS in the SCLC-A and –N subtypes [72]. Furthermore, the SCLC-P subtype was demonstrated to be more sensitive to cisplatin, anti-metabolites, including anti-folates and nucleoside analogues, and PARP-inhibitors (PARPi), despite the modest expression of SLFN11, a key predictor of the response to PARPi according to previous reports [73]. Additionally, the SCLC-N subtype is characterized by high expression of c-MYC, which is a predictive biomarker for the response to Aurora kinase inhibitors (AURKi) [74]. Finally, the SCLC-N and SCLC-I subtypes were demonstrated to be resistant to cisplatin, while the SCLC-A subtype was demonstrated to have a range of sensitivities [72].

Based on these findings, it was hypothesized that non-neuroendocrine tumors express high levels of major histocompatibility complex (MHC)-class I, while NE-SCLC is characterized by the low expression of MHC-class I and low T-cell infiltration, which provide an immunologically “cold” tumor microenvironment, making the tumor less likely to respond to ICIs. Therefore, Fousek et al. investigated an alternative immunotherapy approach, using a natural killer (NK)-based therapy for the treatment of SCLC [75]. Natural killer (NK) cells are a type of cytotoxic lymphocyte belonging to the innate immune system and whose role is analogue to that of cytotoxic T cells. However, unlike cells of the adaptative immune system, NK cells are able to recognize and kill stressed cells in the absence of MHC, allowing for a much faster immune reaction [76]. According to the results of this study, NK cells isolated from healthy donors demonstrated great efficacy in lysing SCLC tumors cells of both the NE subtype (ASCL1- and NEUROD1-positive) and the non-NE subtype (POU2F3- and YAP1-positive). Furthermore, the lytic effect was significantly enhanced by pretreating NK cells with N-803, a clinical-stage super agonist complex of a mutant human IL-15 combined with the sushi domain of hIL15Ra and fused to an IgG1 Fc domain [77]. These findings suggest that NK cells pretreated with N-803 may provide a clinical benefit across all molecular variants of SCLC, including those with a “cold” microenvironment, which are less likely to respond to immunotherapy.

## 6. Other Prognostic and/or Predictive Factors and Data for Long-Term Survivors

PD-L1 expression in SCLC is variable, ranging from 2% to 83% and depending on the assays used [78]. In the IMpower133 trial, the authors conducted an exploratory biomarker analysis on the efficacy of atezolizumab based on the blood-based tumor mutational burden (bTMB) and PD-L1 expression on both tumor cells (TCs) and tumor-infiltrating immune cells (ICs) [16]. Only one third of the patients enrolled had evaluable tumor tissue, and for these patients, the PD-L1 expression level was <1% in TCs in almost all cases (129/137), and the PD-L1 expression level in ICs was <1% in about half of the cases (68/137) [16]. According to this exploratory analysis, HRs for OS favored the addition of atezolizumab to standard chemotherapy across all PD-L1 subgroups [16]. Consistent with these results, the same updated exploratory analysis demonstrated that atezolizumab + EP resulted in improved survival benefit in both high and low bTMB [16].

Similarly, in the CheckMate-032 trial, the authors evaluated the impact of TMB on the survival outcomes of patients treated with nivolumab as a monotherapy or in combination with ipilimumab [10]. This analysis used whole-exome sequencing to determine TMB and divided patients into tertiles, defined as follows: low (<143 mutations), intermediate (143–247 mutations) and high (>248 mutations). According to the results of this analysis, nivolumab + ipilimumab provided a better survival benefit in patients with high TMB when compared to nivolumab alone, whereas for patients with low and medium TMB, no differences in survival were noted with nivolumab plus ipilimumab or nivolumab alone [10].

The phase III CASPIAN trial showed higher PD-L1 expression on ICs than TCs and no significant impact of PD-L1 expression on the effectiveness of treatment, and, similar to the IMpower-133 study, the addition of durvalumab to EP improved OS across all PD-L1 subgroups [79].

In accordance with the analysis of the IMpower133 and CASPIAN trials, in the KEYNOTE-604 study, the use of pembrolizumab in combination with EP was shown to improve the OS and PFS HRs in both PD-L1-positive and -negative subgroups [18]. Conversely, in the phase Ib KEYNOTE-028 trial, patients affected by relapsed SCLC with a combined positive score (CPS, defined as the number of PD-L1-positive cells) ≥1%, who received pembrolizumab, achieved an ORR of 33.3% (95% CI, 16% to 55%) [80]. Additionally, the phase II KEYNOTE-158 trial, investigating the efficacy of pembrolizumab monotherapy, compared patients with a CPS ≥1% with patients characterized by a CPS <1%: in this study, a CPS ≥1% was associated with an improvement in ORRs and mOS compared to a CPS < 1% [81].

Another biomarker that is increasingly studied is the human leukocyte antigen (HLA) system, also known as the human version of the major histocompatibility complex (MHC) [82]. Particularly, HLA class I corresponding to MHC-class I (A, B and C) presents a foreign antigen to CD8+ T-cells, while HLA-class II corresponding to MHC-class II (DP, DQ and DR) is restricted to antigen-presenting cells (APCs), and it is involved in antigen presentation to CD4+ T-cells [82]. Previous in vivo and in vitro studies highlighted that the expression of HLA-class II on TCs influences immune response, cancer progression and tumor immunogenicity [83]. Chen et al. conducted a study with the aim to investigate the association between recurrence-free survival (RFS) and HLA-class II expression on tumor-infiltrating lymphocytes (TILs) and TCs in SCLC [84]. The authors found out that HLA class-II is expressed in only few TCs and in nearly half of TILs. Furthermore, HLA-class II expression on TILs was positively related to longer RFS and negatively related to the presence of lymph node metastases. Nonetheless, RFS was not significantly different in SCLC with HLA-class II expression on TCs [84]. Consistent with these results, Garassino et al. presented a post hoc exploratory analysis of the association between HLA class-I/II and OS in the CASPIAN trial. A total of 52% of patients (414 out of 805) were evaluable for HLA I/II genotyping. Notably, the presence of a particular HLA-class II allele, named DQB1×03:01, was associated with longer OS (14.9 vs. 10.5 months, HR: 0.59) in the durvalumab + tremelimumab + EP arm, but not in the durvalumab + EP arm (14.7 vs. 14.3 months, HR: 0.93) nor in the EP arm (9.7 vs. 10.5 months, HR: 0.94) [85].

Despite the high lethality of SCLC, in both the limited and extensive stages, some patients have demonstrated long-term survival, and some studies have analyzed the clinical and biological characteristics of long-term survivors (LTS) in order to identify potential prognostic factors. Notably, Reinmuth et al. conducted a post hoc analysis aiming to evaluate the clinical characteristics of LTS of the CASPIAN trial (*n* = 94), defined as patients still alive after a median follow-up of 39.4 months [86]. Notably, LTS were three times more abundant in the durvalumab + EP group (16%, *n* = 44) than in the chemotherapy group (5%, *n* = 13). Furthermore, LTS in the durvalumab + EP arm showed a higher incidence of favorable prognostic factors at baseline than the ITT population, such as a lower incidence of liver (20% vs. 40%) and brain metastases (7 vs. 10%). More of the LTS completed the EP induction (≥4 cycles) compared to the ITT population, with greater overall treatment exposure in both the immunotherapy + EP arms. With regard to PD-L1 expression, in the durvalumab + tremelimumab + EP group, only expression ≥1% on TCs or ICs was enriched in patients with an OS ≥ 18 months. This enrichment seemed to be maintained in patients with an OS ≥ 36 months, but data are yet to be presented [86].

Given the interest in LTS, at the time of the IMpower133 study closure, patients treated with atezolizumab were eligible to enroll in the phase IV, single-arm IMbrella A extension and long-term observational study (NCT03148418) [87]. A total of 18 patients were enrolled and the 5-year OS rate was 12%. Of the 11 patients who remain alive and in the study after 5 years, the median age at baseline was 59 years, two patients had baseline BMs and none had baseline liver metastases. The SCLC subtype information was available for 7 out of 11 patients (SCLC-A = 1, SCLC-I = 2, SCLC–N = 4). This long-term follow-up analysis demonstrates that a long-term survival benefit up to 5 years is possible in a subset of patients with SCLC, whose baseline characteristics have to be further investigated.

To identify the biomarkers of LTS, Muppa et al. conducted a case–control study comparing surgically resected tumors from long-term survivors (survival >4 years) and tumors from patients with the expected survival time (<2 years) [88]. According to the results of this study, LTS specimens were characterized by higher concentrations of TILs, monocytes and macrophages, suggesting that a “hot” tumor microenvironment may be a good prognostic factor. Another study investigated TILs subsets (CD3+, CD8+, CD45RO+, FOXP3+ and PD-1+) and the expression of PD-L1 on 32 SCLC BM specimens and four matched primary tumor specimens [89]. The authors found out that the presence of CD45RO+ memory T-cells and PD-L1+ TILs in BMs was associated with more favorable survival outcomes. Particularly, patients with an infiltration of CD45RO+ TILS had a significantly longer mOS (11 months; 95% CI: 0.000–26.148) than patients without CD45RO+ TILs (5 months; 95% CI: 0.966–9.034; *p* = 0.007). Furthermore, PD-L1 expression on TILs was found in 8 out of 32 patient specimens (25.0%), and on tumor infiltrating macrophages, in 9 out of 32 patient specimens (28.1%), and patients with PD-L1 expression on TILs showed an improvement in survival prognosis (29 months vs. 6 months, *p* = 0.002) [89].

## 7. Conclusions

Despite the recent advances in research, SCLC is still considered a lethal and aggressive disease characterized by high metastatic potential. The addition of ICIs to platinum-based chemotherapy resulted in a clinically modest prolongation of overall survival. In addition, differently from other solid tumors where PD-L1 and TMB seem to be potential predictive biomarkers of the response to ICIs, the results from the phase III trials suggest that neither PD-L1 expression nor TMB appear to be suitable biomarkers able to predict responses to chemoimmunotherapy in SCLC. The identification of four SCLC subtypes—characterized by the differential expression of transcription factors (ASCL1, NEUROD1 and POU2F3) or low expression of all three transcription factor signatures accompanied by an inflamed gene signature (SCLC-A, N, P and I, respectively)—could lead to a better understanding of SCLC disease and could provide the right direction for more personalized treatment. The role of radiation therapy—as a consolidation treatment for thoracic disease as well as its prophylactic role in order to prevent BMs— still has to be clarified in patients receiving new chemoimmunotherapies.

In conclusion, the phase III trials investigating the addition of ICIs to platinum–based chemotherapy showed a modest improvement in OS, and studies concerning predictive markers of immunotherapy efficacy are lacking. In this regard, the way toward more effective and personalized treatment for SCLC is still long and perhaps unknown, and immunotherapy could represent a good opportunity to change the landscape of this neglected tumor.

## Figures and Tables

**Table 1:** Phase III ES-SCLC trials.

	IMpower 133	CASPIAN	KEYNOTE-604	CAPSTONE-01	ASTRUM-005
Treatment arms	Atezolizumab + CE Q3W × 4 cycles	Durvalumab + tremelimumab + EP Q3W × 4 cycles	Pembrolizumab + EP Q3W × 4 cycles	Adebrelimab + CE Q3W × 4/6 cycles	Serplulimab + CE Q3W × 4 cycles
		Durvalumab + EP Q3W × 4 cycles	Placebo + EP Q3W × 4 cycles	Placebo + CE Q3W × 4/6 cycles	Placebo + CE Q3W × 4 cycles
	Placebo + CE Q3W × 4 cycles	EP Q3W × 6 cycles			
IO maintenance	Yes	Yes	Yes	Yes	Yes
mOS HR (*p*-value)	12.3 vs. 10.3 mo 0.70 (0.007)	12.9 vs. 10.5 mo (D + CT vs. CT) 0.71 (0.0003)	10.8 vs. 9.7 mo 0.80 (0.0164)	15.3 vs. 12.8 mo 0.72 (0.0017)	15.4 vs. 10.9 mo 0.63 (<0.001)
		10.4 vs. 10.5 mo (D + T + CT vs. CT) 0.81 (0.0200)			
mPFS HR (*p*-value)	5.2 vs. 4.3 mo 0.77 (0.02)	5.1 vs. 5.4 mo (D + CT vs. CT) 0.80 N/A	4.5 vs. 4.3 mo 0.75 (0.0023)	5.8 vs. 5.6 mo 0.67 (<0.0001)	5.7 vs. 4.3 mo 0.48 NR
		4.9 vs. 5.4 mo (D + T + CT vs. CT) 0.84 N/A			
ORR	60.2%	68% (D + CT)	70.6%	70.4%	80.2%
		58% (D + T + CT)			
% BMs	8.5%	10% (D + CT)	14.5%	2%	12.9%
% LM	38.3%	39% (D + CT)	41.7%	32%	25.4%
BMs OS HR	0.96	0.76	1.32	NR	0.61
LM OS HR	0.75	0.87	0.75	0.92	NR
PCI	Allowed	Only allowed in the CT arm	Allowed	Allowed at least 14 days before the first dose	Not specified
Consolidation RT	Not allowed	Not allowed	Not specified	Not allowed	Not specified
% TR grade 3–4 AEs	57.1%	46%	63.7%	86%	82.5%
% TR grade 5 AEs	1.5%	4.30%	2.7%	1%	7.7%
% Age ≥ 65	44.8%	38%	49.6%	33%	60.4%
PD-L1	<1%: 47.4% ≥1%: 52.6%	<1%: 95% ≥1%: 5%	<1%: 42.5% ≥1%: 38.6%	<1%: 85% ≥1%: 10%	<1%: 83.6% ≥1%: 16.4%

CE = carboplatin + etoposide; EP = platinum + etoposide, IO = immunotherapy; N/A = not applicable; NR = not reported; mOS = median overall survival; ORR = objective response rate; mo = months; CT = chemotherapy; RT = radiotherapy; BMs = brain metastases; LM = liver metastases; PCI = prophylactic cranial irradiation; TR = treatment-related; AEs = adverse events; Q3W = every 3 weeks; PD-L1 = programmed death ligand 1.

**Table 2:** Ongoing trials.

	ACHILES	ADRIATIC	RAPTOR	SKYSCRAPER-02
Disease stage	LS-SCLC	LS-SCLC	ES-SCLC	ES-SCLC
Study phase	II	III	II/III	III
Treatment arms	Atezolizumab Q3W	Durvalumab + tremelimumab Q4W	Atezolizumab Q3W	Tiragolumab + atezolizumab + CE Q3W
		Durvalumab + placebo Q4W	Atezolizumab Q3W + RT QD for 5 weeks	Placebo + atezolizumab + CE Q3W
	Observation	Placebo + placebo Q4W		
PCI	Not specified	Allowed	Allowed	Not specified
Primary endpoints	2-year survival	PFS and OS	PFS and OS	PFS and OS

S-SCLC = limited-stage small-cell lung cancer; ES-SCLC = extensive-stage small-cell lung cancer; CE = carboplatin + etoposide; Q3W = every 3 weeks; Q4W = every 4 weeks; RT = radiotherapy; QD = every day; PCI = prophylactic cranial irradiation.

**Table 3:** Randomized studies evaluating thoracic radiotherapy in ES-SCLC after first-line chemotherapy.

Reference	Study Design	Patients	RT dose	Results
Jeremic et al. 1999 [41]	Phase II	210	54 Gy	OS at 5 years 9.1% (RT arm) vs. 3.7% (no RT arm) Median OS 17 (RT arm) vs. 11 months (no RT arm)
Narayan et al. 2015 [42]	Phase III	358	45 Gy	Median PFS 15 months (RT arm) vs. 10 months (no RT arm) OS at 5-years 10.3% (RT arm) versus 6.2% (no RT arm)
Slotman et al. 2015 [39]	Phase III	498	30 Gy	OS at 1 year 33% (RT arm) vs. 28% (no RT arm) OS at 2 years 13% (RT arm) vs. 3% (no RT arm)
Gore et al. 2017 [40]	Phase II	97	30/45 Gy	OS at 1-year 50.8% (RT arm) vs. 60.1% (no RT arm Longer time to progression in RT arm

**Table 4:** Ongoing studies evaluating SRS for SCLC brain metastases.

Trial	Allocation/Phase	Arm	Primary End Point	Notable Secondary End Points	Estimated No. of Patients	Start Date– Estimated End
Whole Brain Radiation Therapy Alone vs. Radiosurgery for SCLC Patients With 1–10 Brain Metastases (ENCEPHALON)	Randomized/NA	Arm A: SRS Arm B: WBRT	Neurocognition	Intracranial progression (number or dimension) OS PFS QoL	56	December 2017–October 2024
A Study of Stereotactic Radiosurgery (SRS) for People With Lung Cancer That Has Spread to the Brain	Phase 2	SRS	OS	-	62	June 2022–June 2025
Stereotactic Radiosurgery for the Treatment of Patients With Small-Cell Lung Cancer Brain Metastasis	Phase 2	SRS	Cognitive decline	OS PFS LC	50	August 2020–December 2024
Testing if High Dose Radiation Only to the Sites of Brain Cancer Compared to Whole Brain Radiation That Avoids the Hippocampus is Better at Preventing Loss of Memory and Thinking Ability	Randomized/Phase 3	Arm I (SRS) Arm II (HA-WBRT, memantine)	Time to Neurocognitive Failure	OS Time to Neurologic Death	200	March 2021–July 2030
Stereotactic Radiation in Patients With Small-Cell Lung Cancer and 1–10 Brain Metastases	Phase 2	SRS	Death due to progressive neurologic disease	QoL Neurocognitive function Radionecrosis LC	100	February 2018–June 2025
